# The Effects of Legume Consumption on Markers of Glycaemic Control in Individuals with and without Diabetes Mellitus: A Systematic Literature Review of Randomised Controlled Trials

**DOI:** 10.3390/nu12072123

**Published:** 2020-07-17

**Authors:** Dale Bielefeld, Sara Grafenauer, Anna Rangan

**Affiliations:** 1 Nutrition and Dietetics Group, School of Life and Environmental Sciences at the Charles Perkins Centre, The University of Sydney, Johns Hopkins Dr, Camperdown, NSW 2006, Australia; dbie0493@uni.sydney.edu.au (D.B.); anna.rangan@sydney.edu.au (A.R.); 2 Grains & Legumes Nutrition Council, Mount Street, North Sydney 2060, Australia; 3 School of Medicine, University of Wollongong, Northfields Avenue, Wollongong, NSW 2522, Australia

**Keywords:** legumes, Fabaceae, glycaemic control, diabetes mellitus, metabolic syndrome, insulin resistance

## Abstract

Legumes are a rich source of dietary fibre, plant protein, and low-Glycaemic Index (GI) carbohydrate. Evidence suggests a positive effect on glycaemic control following a single meal; however, the effects of habitual consumption are less clear. This review aimed to investigate whether medium-to-long-term legume consumption had an effect on markers of glycaemic control in individuals with diabetes mellitus, without diabetes mellitus, or with prediabetes. As per the Preferred Reporting Items for Systematic Reviews and Meta-Analyses (PRISMA) protocol, the online databases MEDLINE, Embase, CENTRAL, and CINAHL were searched from inception through to 31 March 2020. Randomised controlled trials (RCTs) ≥6 weeks in duration, reporting ≥1 of the following: fasting blood glucose (FBG), fasting blood insulin (FBI), glycosylated haemoglobin (HbA1c), homeostatic model assessment-insulin resistance (HOMA-IR), or 2-h postprandial glucose (2-h PPG), were deemed eligible. The overall quality of evidence was determined using the Grading of Recommendations Assessment, Development, and Evaluation (GRADE) assessment. A total of 18 RCTs were included, of which, 5 focused on individuals with diabetes mellitus, 12 on individuals without diabetes mellitus, and one on individuals with prediabetes. Only studies of those with type 2 diabetes mellitus (*n* = 5) reported significant effects for legume interventions, three of which consistently reported reductions in FBG, two reported reductions in HbA1c, one reported a reduction in FBI, and another a reduction in 2-h PPG (*p* < 0.05); however, the overall quality of evidence was very low. The findings of this review support the dietary inclusion of legumes; however, the need for further high-quality RCTs to be conducted is also highlighted, particularly among individuals with prediabetes, gestational diabetes mellitus and type 1 diabetes mellitus.

## 1. Introduction

Legumes, as defined by the Food and Agriculture Organisation (FAO), are derived from the botanical family Fabaceae (or Leguminosae) and include chickpeas, lentils, beans, peas, and dried pulses [1]. Legumes are unique foods with a nutrient-rich profile comprising of iron, zinc, potassium, magnesium, niacin, dietary fibre, and a particularly rich source of ecologically sustainable protein [2,3]. Notably, legumes are also considered a low glycaemic index (GI) food, effective at reducing the postprandial glucose and insulin response compared to that of other carbohydrate-containing foods, such as rice or potatoes [3]. The health-promoting qualities elicited by legumes are well documented, including improved metabolic health [4], reduced risk of coronary heart disease [5], and reduced risk of all-cause mortality [6], and as such are featured in national healthy eating guidelines around the world [2,7,8,9]. Foundation diet modelling has, however, found Australia’s consumption to be suboptimal, suggesting current consumption would need to increase by 470% to meet recommended nutritional targets [10]. 

Given the health trajectory of the population, with approximately 382 million people currently living with diabetes mellitus, and projected estimates anticipating an increase to 592 million people by 2035 [11], we proposed that regular consumption of high-fibre low-GI legumes may be of benefit. In Australia, diabetes mellitus has been identified as one of the most rapidly increasing chronic diseases [12], with an approximate 21% of the population currently living with some degree of impaired glucose metabolism, presenting as type 1 diabetes mellitus (T1DM), type 2 diabetes mellitus (T2DM), or prediabetes [12,13]. In addition, 6.9−13.6% of all pregnancies within Australia are affected by gestational diabetes mellitus (GDM), posing an increased risk in health complications for both mother and child [14]. As such, diabetes mellitus has been recognised as a national health priority [14]. A growing body of research exists investigating the potential role of legumes to support metabolic health; however, the effects of habitual legume consumption, particularly within population groups of varying degrees of glucose metabolism impairment, remain unclear. Further research is warranted to determine the sustainability of the conferred benefits on glycaemic control that have been observed following acute and short-term trials [15]. Therefore, this systematic literature review aimed to investigate whether medium-to-long-term legume consumption had an effect on markers of glycaemic control in individuals with diabetes mellitus, individuals without diabetes mellitus, and individuals with prediabetes. 

## 2. Methods 

This systematic literature review was conducted in accordance with the Preferred Reporting Items for Systematic Reviews and Meta-Analyses (PRISMA) guidelines [16] with the protocol defined prior to database screening and submitted to Prospective Register of Systematic Reviews (PROSPERO) (Registration ID: CRD42020179734).

### 2.1. Eligibility and Exclusion Criteria

The research question ‘Is there an effect of legume consumption on markers of glycaemic control in individuals with diabetes mellitus, individuals without diabetes mellitus, or individuals with prediabetes?’ was developed using the Population, Intervention, Intervention, Outcome (PICO) format (Table S1). To be included in the review, publications were required to meet the inclusion criteria: (a) Randomised controlled trial (RCT), parallel, or cross-over design; (b) studies conducted in humans aged ≥18 years including individuals with diabetes mellitus, including T1DM, T2DM, or GDM, individuals without diabetes mellitus, or individuals with prediabetes, regardless of medication use or presence of comorbidities; (c) studies with legume-only interventions including chickpeas, beans (kidney, pinto, black, cannellini, white, fava, adzuki, borlotti, flageolet, lima, mung), peas (black-eyed, blue, maple, white, dun), lentils (green, red, yellow, French), lupin, or non-oil seed pulses; (d) reporting ≥1 of the following markers of glycaemic control: fasting blood glucose (FBG), fasting blood insulin (FBI), glycosylated haemoglobin, % value of total haemoglobin (HbA1c), homeostatic model assessment-insulin resistance (HOMA-IR), 2-h postprandial glucose (2-h PPG); and (e) study duration ≥6 weeks, to capture the minimum duration of time that HbA1c, a primary marker of medium-to-long-term glycaemic control, may be expected to change [17]. 

The following exclusion criteria applied: (a) Studies with a population focus on diabetes insipidus; (b) study intervention arms not randomised; (c) studies focusing on peanuts, soybeans, or soy products (e.g., tofu, edamame beans, or soymilk) were excluded due to the differing nutritional profile compared to that of non-oil seed pulses; (d) study interventions with dietary patterns that encompass legumes (e.g., Mediterranean diet) or legume consumption as part of a vegetable intervention; and (e) legume provision in the form of a powder or extract, i.e., not in whole form.

### 2.2. Search Strategy

The following online databases were searched: MEDLINE, Embase, Cochrane Central Register of Controlled Trials (CENTRAL) (via https://ovidsp.ovid.com/), and CINAHL (via https://www.ebsco.com/) from inception up until 31 March 2020. In addition, reference lists of eligible studies were scanned and Pubmed (https://pubmed.ncbi.nlm.nih.gov/) was searched manually for additional studies. See Table S2 for search terms and Boolean operators. No language or date restrictions were applied to the search strategy.

### 2.3. Study Selection, Data Extraction, and Quality Assessment

Reviewer D.B. extracted all retrieved citations into Endnote X9, with duplicates removed using the inbuilt function. Reviewer D.B. independently double screened all titles and abstracts, with any uncertainty resolved with assistance from researchers A.R. and S.G. Following title and abstract screening, a full-text screen was completed on the remaining articles by two independent reviewers (D.B. and S.G.). Reviewers met and resolved any discrepancies, with any remaining uncertainty resolved by a third reviewer (A.R.).

A data extraction form was created in Microsoft Excel to facilitate retrieval and storage of relevant data. Extracted data included study citation, study design (parallel or cross-over), wash-out period (cross-over studies only), study duration, participant characteristics (including diabetes status and class, co-morbidities, Body Mass Index (BMI), age), number of participants including number of males and females, medication usage, legume type and dose (g/day), control diet characteristics, outcomes measured, and results obtained (baseline and endpoint data, and reported *p*-value). Reviewer D.B. contacted the authors of any studies for any data that was absent.

The included studies were assessed for within-study risk of bias using the revised Cochrane risk-of-bias (RoB) tool for randomised controlled trials [18]. Reviewer D.B. assessed studies to determine whether each study had low, some concerns, or high risk of bias. Assessment criteria included risk of bias arising from the randomisation process, deviations from intended interventions, missing outcome data, measurement of the outcome, or selection of the reported result [18]. Any uncertainties were resolved by consultation with a second reviewer (S.G.). The Grading of Recommendations Assessment, Development, and Evaluation (GRADE) assessment was used to assess the overall quality of evidence for each outcome measure within each population group. The quality of evidence was assessed based on risk of bias, inconsistency, indirectness, imprecision, or publication bias, and downgraded where appropriate. Factors for increasing quality of evidence were also considered [19,20]. Quality of evidence was rated as very low, low, moderate, or high.

### 2.4. Data Analysis

A descriptive analysis was conducted based on reported mean ± SD of baseline and endpoint data and statistical significance (*p*-value) for within-group and between-group intervention changes for each study. According to the included studies, outcomes were considered statistically significant when *p* < 0.05. Where required, standard error (SE) and 95% confidence intervals (CIs) were converted to standard deviation (SD) (SD = SE√n) (SD = √n × (upper limit–lower limit)/3.92) using Microsoft Excel [21]. All outcome measures were converted to International System of Units (SI) units (glucose; 1 mg/dL = 0.055 mmol/L), (Insulin; 1 µIU/mL = 6 pmol/L) [22]. Studies were categorised according to population characteristics based on the authors’ description of participants; individuals with diabetes mellitus (T1DM, T2DM, or GDM), without diabetes mellitus, and individuals with prediabetes.

## 3. Results

### 3.1. Search Results and Study Selection

**Figure 1:**
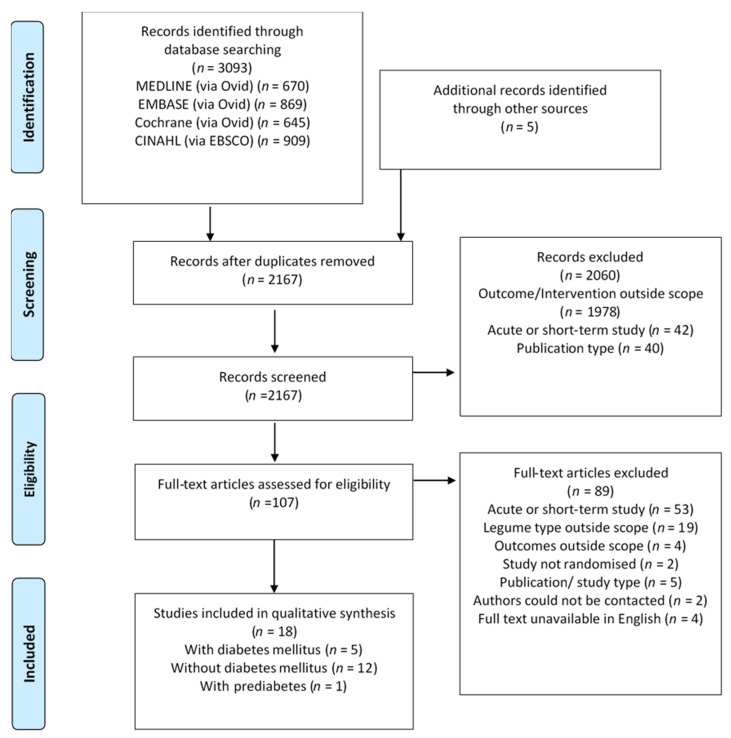
Preferred Reporting Items for Systematic Reviews and Meta-Analyses (PRISMA) flow diagram for study selection.

The initial search, conducted on 31 March 2020, returned a total of 3093 studies. An additional five studies were identified from the reference lists of eligible studies and manual searches on PubMed. The removal of duplicates left 2167 studies to be screened, of which 2060 were excluded based on the title and abstract. A full-text review of the remaining 107 studies resulted in the exclusion of 89 due to the use of legume extract (*n* = 12), study duration <6 weeks (*n* = 53), legume type not eligible or legume not emphasised in intervention (*n* = 7), outcome measures outside scope (*n* = 4), intervention arms not randomised (*n* = 2), publication type unsuitable (*n* = 5), authors could not be contacted (*n* = 2), or full text not available in English (*n* = 4). A remaining total of 18 randomised controlled trials met the inclusion criteria and were hence included in the qualitative synthesis (Figure 1).

### 3.2. Study Characteristics

Five studies, comprising of six legume intervention comparisons, were included for individuals with diabetes mellitus. A total of five legume comparisons had a focus on individuals with T2DM, one on individuals with T1DM, and none on women with GDM. The studies had a total of 251 participants, a mean duration of 8 weeks (range: 6–13 weeks), and a mean legume dose of 100 g/day (range: 50–190 g/day), with one study not reporting the legume dose (Table 1). A total of 12 studies were included for individuals without diabetes mellitus. The studies had a total of 605 participants, a mean duration of 9.5 weeks (range: 6–16 weeks), and a mean legume dose of 164 g/day (range: 81–285 g/day), with two studies not reporting the legume dose (Table 2). One study, comprising of two legume intervention comparisons, was included for individuals with prediabetes. The study had 16 participants, a duration of 6 weeks, and a legume dose of 90 g/day (Table 3).

### 3.3. Risk of Bias

**Figure 2:**
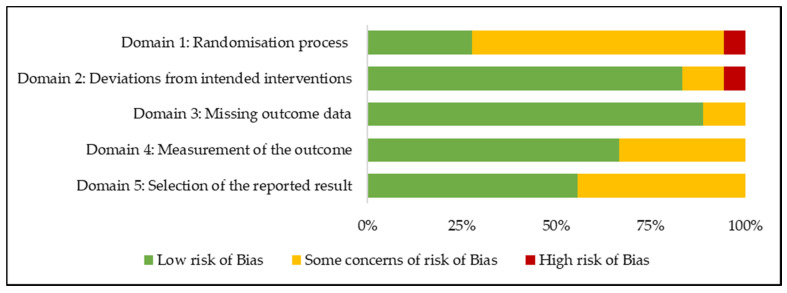
Risk of bias assessment using the revised Cochrane risk-of-bias (RoB) tool in 18 randomised controlled trials examining the effects of legume consumption on markers of glycaemic control.

The results of the within-study risk of bias assessment are summarised in Figure 2. The included studies were assessed according to the predefined criterion outlined in the revised Cochrane RoB 2 tool for randomised controlled trials [18]. According to the Domain 1: Randomisation process, one study had a high risk of bias, with the remaining studies rated as low or some concerns. According to Domain 2: Deviations from intended intervention, one study had a high risk of bias, with the remaining studies rated as low or some concerns. All studies were rated as having a low risk of bias or some concerns of risk of bias according to Domain 3: Missing outcome data, Domain 4: Measurement of the outcome, and Domain 5: Selection of the reported result. Overall, studies were rated as having a low risk of bias (*n* = 4), some concerns for risk of bias (*n* = 12), or a high risk of bias (*n* = 2) (Figure S1). 

### 3.4. GRADE Assessment

The GRADE assessment was completed for two outcome measures among individuals with T2DM: FBG and HbA1c, as the evidence base for these outcome measures comprised of three or more studies. The quality of evidence for these outcomes was downgraded based on indirectness, imprecision, and publication bias, and overall determined to be very low (Table 4). The quality of evidence could not be increased based on the magnitude of effect, dose–response gradient, or effect of plausible residual confounding for any outcome measures [42]. The quality of evidence could not be assessed for FBI and 2-h PPG for individuals with T2DM, 2-h PPG for individuals without diabetes mellitus, or any outcome measures for individuals with T1DM or prediabetes due to the limited evidence base (number of studies < 3) or raw data not reported by authors. The quality of evidence for all outcomes (FBG, FBI, HOMA-IR, and HbA1c) for individuals without diabetes mellitus were also found to be very low (Table S3).

### 3.5. Effect of Intervention on the Outcome

#### 3.5.1. Individuals with Diabetes Mellitus 

Among the five studies assessing the effects on individuals with T2DM, several reported statistically significant effects on markers of glycaemic control following legume consumption (Table 5). While only one study was identified that assessed the effects on individuals with T1DM, there was limited evidence to suggest whether or not legumes had an effect. No studies were identified in those with GDM; therefore, no results can be reported. Three studies (T2DM *n* = 3) observed a statistically significant between-group effect on FBG in favour of the legume intervention, with reductions ranging from 0.13−1.59 mmol/L, *p* < 0.05 [24,25,26]. Two studies (T2DM *n* = 2) reported a statistically significant between-group effect on FBI in favour of the legume intervention [23,24]; however, one of these also reported a statistically significant between-group difference at baseline *(p =* 0.02) [23]. Three studies (T2DM *n* = 3) observed a statistically significant between-group reduction in HbA1c in favour of legume intervention, with reported reductions ranging from 0.10−0.50% [23,25,27], however, one study also reported a statistically significant between-group difference at baseline (*p =* 0.04) [23]. One study, comprising of two comparisons (T2DM *n* = 1, T1DM *n* = 1), reported statistically significant between-group effects for 2-h PPG in favour of legume interventions; however, raw baseline data was not reported and therefore the absolute change is unknown [27]. This was the only statistically significant effect observed in individuals with T1DM. No studies compared the effects of HOMA-IR in individuals with diabetes mellitus.

#### 3.5.2. Individuals without Diabetes Mellitus

The 12 identified RCTs conducted on individuals without diabetes mellitus reported inconsistent results, albeit no between-group effects observed were statistically significant. Two studies reported a statistically significant within-group decrease in FBG for the legume intervention [29,35]; however, one of these also reported a statistically significant within-group decrease in the control intervention arm [35] (Table 6). One study reported statistically significant within-group decreases in FBI for both the control and legume interventions (*p* < 0.01), and a 12-month follow-up also reported a significant within-group reduction in both the intervention and control groups (*p* < 0.02) [35] (Table 7). One study observed a statistically significant within-group decrease in HOMA-IR for the legume intervention (*p* < 0.001) [35], and another observed a statistically significant within-group decrease in HbA1c for the legume intervention (*p* = 0.01) [36]; however, both studies also observed a statistically significant effect in control arms. One study reported a statistically significant between-group effect for 2-h PPG (*p* = 0.01) in favour of the control [36], and another study reported a statistically significant within-group decrease (*p* < 0.0001) in both the control and intervention arms [35] (Table S4).

#### 3.5.3. Individuals with Prediabetes

The results of this systematic review found limited evidence to suggest whether or not legume consumption had an effect on markers of glycaemic control in individuals with prediabetes. Only one study, comprising of two different legume intervention comparisons, being pinto beans and black-eyed peas, reported on FBG, FBI, HOMA-IR, and HbA1c and found no statistically significant between-group or within-group effects [41]. 

## 4. Discussion

Regular legume consumption may play a considerable role in reducing the risks associated with T2DM. Improvements in glycaemic control were consistently observed among legume interventions for individuals with T2DM within several studies identified by this review. Three studies observed reductions in FBG, two studies observed reductions in HbA1c, one observed a reduction in FBI, and another observed a reduction in 2-h PPG. Legume interventions were all in line with the acceptable macronutrient distribution ranges (AMDRs) for carbohydrate, fat, and protein [44] and encompassed a variety of legume types, such as chickpeas, lentils, peas, and a variety of beans. All control diets advised participants to avoid or minimise consumption of legumes. Included studies were at least six weeks in duration; however, as per the GRADE assessment, the quality of evidence was found to be very low. 

The observed 0.10−0.50% reductions in HbA1c among legume interventions for individuals with T2DM were consistent with previously reported results by Sievenpiper et al. [15], who observed a ~0.48% reduction. It must, however, be noted that the shortest included trial by Sievenpiper et al. was only two weeks in duration, meaning the results are not directly comparable to the results from this review. In individuals without diabetes mellitus, nor any degree of glucose metabolism impairment, internal homeostatic mechanisms effectively regulate glycaemia and, as anticipated, no significant effects were identified by this review [45]. In contrast, Sievenpiper et al. [15] previously found legumes to have a decreasing effect on FBG among individuals without diabetes mellitus; however, considerable heterogeneity was identified in the included studies. Despite anticipating some effects on glycaemic control following legume consumption among individuals with prediabetes, none were observed. However, this may have been due to the diet prescription applied to the control participants, which would also be considered high in dietary fibre, with a well-controlled energy and macronutrient profile. 

Despite the consistently significant effects observed between studies for individuals with T2DM, differences between interventions must be acknowledged. The use of medication within this population group was identified as being a potential confounder and therefore transparency in reporting and the analysis of subgroups has been highlighted as an important consideration. Hosseinpour-Niazi et al. [24] and Jenkins et al. [25] exclusively recruited participants who had been prescribed a stable dose of oral glucose-lowering agents for at least 3 months or 2 months, respectively. Shams et al. [26] excluded participants on insulin therapy, and there were no reports of use of other oral glucose-lowering agents. The earliest study by Simpson et al. [27] did not report a minimum time period for the use of relevant medication for participants, nor was subgroup analysis conducted to identify the effect of insulin use in those with T1DM. This is a significant weakness of this study. 

Among individuals without diabetes mellitus, inconsistencies between study designs were also identified. Despite individuals being normoglycaemic, variations among participant characteristics within our review existed including obesity [36], hypercholesterolemia [40], or polycystic ovarian syndrome (PCOS) [35]. Furthermore, control interventions varied between studies, some being carrots [40], wheat-based foods [37], or low-carbohydrate diets [39]. Therefore, control diets were not directly comparable between studies, and this issue had the potential to minimise differences observed between intervention and control arms within studies, or enhance effects observed for legume interventions. This was particularly the case where dietary fibre intake was not matched with control [29,30,32,33,34,35,36,37,38,39,40] or macronutrient energy contribution was not matched [39]. In addition, some studies were hypocaloric and achieved weight loss among overweight and obese participants [29,32,34]. Legume interventions showed greater weight reductions compared with hypocaloric control diets, suggesting hypocaloric diets containing legumes may be more effective for weight loss than a conventional diet [14,28,46].

A proposed mechanism by which legumes may aid in the management of T2DM is via the rich soluble and insoluble dietary fibre content. Soluble dietary fibre has been shown to reduce peak blood glucose via increased luminal content viscosity, while several mechanisms have been proposed for insoluble dietary fibre, including modulation of the release of gastric hormones and a delayed absorption of monosaccharides [47]. An increased consumption of intrinsic dietary fibre has been shown to elicit significant improvements in FBG, FBI, and HbA1c among individuals with diabetes mellitus [14,17], consistent with the results from this review. 

Dietary fibre intakes in intervention arms were in line with the adequate intake (AI) as per the Australian nutrient reference values (25 g for women, 30 g for men) [30,32,34,35,36,39,44], with the exception of four study interventions, which were not [23,33,41]. Simpson et al. [27] reported an extreme 96 g/day dietary fibre intake, which would not be sustainable among free-living individuals in the longer term. The serving size of legumes also varied considerably within study intervention designs, ranging from 50−190 g/day of cooked legumes, with significant effects being observed across the entire dose range. Notably, serving sizes varied considerably in comparison to the proposed international minimum target recommendation of 100 g/day suggested by Marinangeli et al. [2] as well as the proposed target set by the Grains and Legumes Nutrition Council, being 100 g at least three times per week [48].

The acceptability and tolerability of regular legume consumption was considered by six studies [25,30,33,35,36,39] and adverse gastrointestinal symptoms were experienced by 83 out of a total of 320 participants. These included abdominal pain, flatulence, bloating, or altered bowel habits. All adverse events were rated as being mild to moderate in severity, with none rated as serious. Given the high dietary fibre content of legumes, and the presence of fermentable carbohydrates, it is not unexpected that the increase in legume consumption caused some discomfort among participants. Notably, Tonstad et al. [39] and Gravel et al. [33] both included acclimatisation periods of three and four weeks, respectively, prior to the intervention. This allowed a gradual increase in the amount of legumes and total dietary fibre consumed. Despite beans being included in all meals, and a more than doubling of the dietary fibre intake compared to the control, Tonstad et al. [39] reported that the intervention was well tolerated. This perhaps suggests the importance of gradually increasing legume consumption to minimise potential gastrointestinal upset, which is also recommended in the clinical setting. 

### 4.1. Strengths and Limitations

Several strengths and limitations have been acknowledged within this systematic literature review. A strength was the distinction between population groups based on the degree of glucose metabolism impairment, allowing for a more specific understanding of the effects of legumes, and the inclusion of co-morbidities ensured results were still generalisable. This systematic literature review searched databases from inception inclusive to the date of extraction, and as a result, the included study publication dates spanned from 1981 through until 2019, allowing high-quality RCTs to be captured regardless of publication date. Implementation of the Cochrane risk of bias tool and the GRADE assessment allowed any limitations within studies, and the overall evidence base, to be identified and acknowledged. Limitations of this review arise from possible publication bias as grey literature sources were not included, nor were studies published in languages other than English. Furthermore, included studies had relatively small sample sizes, and given databases were searched from inception, the reporting style of some studies was poor. Assessment of the intervention effects was also made more difficult due to confounding factors, such as medication use, variations within participant populations, and variations within trial protocols, and as such the evidence was found to be of very low quality according to the GRADE assessment, meaning the estimate of the effect is very uncertain. The evidence obtained by this review is limited in that a pooled power of effect could not be estimated.

### 4.2. Future Directions

To progress research in this area, we suggest future RCTs consider computer-generated randomisation methods with sufficient measures in place for double blinding, where possible. A minimum trial duration of 12 weeks is recommended to observe accurate changes in HbA1c. Diet prescriptions in both arms of RCT should be isocaloric and ensure that dietary fibre intake and macronutrient composition are maintained, limiting differences between control and intervention arms. Additionally, the legume serving size provided must be carefully considered. The studies identified by this review observed statistically significant effects following doses ranging from 50−190 g/day, with no serious adverse events reported; therefore, future trials may consider using similar doses. Focus should be placed on population groups for which the evidence base is particularly limited, in those with prediabetes and GDM, as well as for individuals with T1DM whose glycaemic control tends to be poor. 

## 5. Conclusions

This systematic literature review was the first to our knowledge to exclusively examine whether medium-to-long-term legume consumption has an effect on markers of glycaemic control in individuals with and without diabetes mellitus. Several studies identified by this review found regular legume consumption, for a duration of at least six weeks, to have statistically significant effects among individuals with T2DM; however, the evidence base was considered to be very low quality. The findings of this review support regular dietary inclusion of legumes; however, the body of research for individuals with prediabetes, GDM, and T1DM is extremely limited, suggesting the need for further high-quality longer term RCTs to be conducted.

## Figures and Tables

**Table 1:** Characteristics of studies examining legume consumption in individuals with diabetes mellitus.

Study	Design ^a^ and Duration	*n* (I/C) ^b^	Characteristics ^c^	(M/F) ^d^	Age (Years)	Anti-Diabetic Medication ^e^	Legume Type ^f^ Dose (g/day) ^g^ Control	%E (CHO: Fat: Pro) ^h^ Dietary Fibre ^i^ (g/day)	Energy Balance
Hassanzadeh-Rostami et al. 2019 [23]	P 8 wks	(20/23)	T2DM, BMI I: 27.3 ± 3.4 C: 26.5 ± 3.2	(13/32) ^#^	I: 59.6 ± 6.0 C: 56.1 ± 7.2	Yes	Legume: NR 77 Control: Legume free	I: 52:32:17 C: 56:29:16	Isocaloric
								I: 17.3 ± 4.7 C: 17.2 ± 4.7	
Hosseinpour-Niazi et al. 2015 [24]	C 8 wks	31	T2DM, BMI: I: 27.7 ± 3.3 C: 27.8 ± 3.3	(7/24)	58.1 ± 6.0	Yes, ≥3 months	Legume: Mixed (L, CP, B, P) 83 Control: Legume free	I: 54:32:14 C: 52:34:15	Isocaloric
								I: 31.4 ± 8.4 C: 26.9 ± 7.2	
Jenkins et al. 2012 [25]	P 13 wks	(60/61)	T2DM, BMI: I: 31.4 ± 7 C:29.9 ± 5.5	(61/60)	I: 58.0 ± 10.1 C: 61.0 ± 7.8	Yes, ≥2 months	Legume: Mixed (L, CP, B) 190 Control: Wheat-based diet	I: 45:31:23 C: 48:29:21	Isocaloric
								I: 39.4 ± 13.1 C: 26.9 ± 5.2	
Shams et al. 2010 [26]	C 6 wks	30	T2DM, BMI: 28.9 ± 4.1	NR	50.2 ± 3.8	NR	Legumes: Lentils 50 Control: Legume free	I: 48:31:18 C: 53:28:20	Isocaloric
								I: 28.6 ± 3.4 C: 23.3 ± 6.4	
Simpson et al. 1981 [27]	C 6 wks	18	T2DM BMI: NR	(10/8)	52.5 ± 12.3	Yes, (*n* = 15)	Legumes: Beans (Mixed) NR Control: Low CHO	I: 61:18:21 C: 40:39:21	Isocaloric
								I: 96.6 C: 17.6	
Simpson et al. 1981 [27]	C 6 wks	9	T1DM BMI: NR	(4/5)	41.2 ± 14.8	Insulin (*n* = 9)	Legumes: Beans (Mixed) NR Control: Low CHO	I: 61:18:21 C: 40:18:21	Isocaloric
								I: 96.6 C: 17.6	

**Abbreviations:** Not Reported (NR); Weeks (wks); Grams per day (g/day); ^a^ Parallel (P), Cross-over (C); ^b^ Number of participants (*n*), Intervention (I), Control (C); ^c^ Type 2 Diabetes Mellitus (T2DM), Type 1 Diabetes Mellitus (T1DM), Body Mass Index (BMI) reported as kg/m^2^; ^d^ Male (M), Female (F); ^e^ Use of Metformin and/or other agents for glycaemic control; ^f^ Lentils (L), Chickpeas (CP), Beans (B), Peas (P); ^g^ Reported as wet weight (1 g dry weight = 2.75 g wet weight, 1 mL = 0.76 g) [28]; ^h^ Macronutrient energy contribution (%E) (Carbohydrates: Fat: Protein); ^i^ Endpoint dietary fibre intake for legume intervention, ^#^ Baseline data; endpoint NR.

**Table 2:** Characteristics of studies examining legume consumption in individuals without diabetes mellitus.

Study	Design ^a^ and Duration	*n* (I/C) ^b^	Characteristics ^c^	(M/F) ^d^	Age (Years)	Anti-Diabetic Medication ^e^	Legume Type ^f^ Dose (g/day) ^g^ Control	%E (CHO: Fat: Pro) ^h^ Dietary Fibre ^i^ (g/day)	Energy Balance
Abete et al. 2009 [29]	P 8 wks	(8/10)	Obese, BMI: 31.8 ± 4.1	(18/0)	38.0 ± 7.0	NR	Legumes: NR NR Control: No legumes	I: 52:30:18 C: 51:33:19	Hypocaloric
								I: 26.5 ± 15.3 C: 20.3 ± 17.1	
Abeysekara et al. 2012 [30]	C 8 wks	87	BMI: 27.5 ± 4.5	(30/57)	59.7 ± 6.3	(*n* = 3)	Legumes: Mixed (L, CP, B, P) 250 Control: Usual diet	I: 48:37:15 C: 47:38:16	Isocaloric
								I: 30.0 ± 15.0 C: 22.0 ± 10.0	
Alizadeh et al. 2014 [31]	P 6 wks	(17/17)	WC > 88 cm BMI: NR	(0/34)	36.1 ± 8.2	Nil	Legumes: Mixed (L, CP, P, B) 190 Control: No legumes	I: 55:30:15 C: 55:30:15	Hypocaloric
								NR	
Crujeiras et al. 2007 [32]	P 8 wks	(15/15)	Obese, BMI: 32.0 ± 5.3	(17/13)	36.0 ± 8.0	Nil	Legumes: Mixed (L, CP, B, P) NR Control: No legumes	I: 50:33:19 C: 51:31:19	Hypocaloric
								I: 25.0 ± 6.0 C: 18.0 ± 5.0	
Gravel et al. 2010 [33]	P 16 wks	(60/54)	2 risk factors for MetSyn, BMI: I: 29.6 ± 4.5 C: 30.1 ± 5.7	(0/114)	I: 52.5 ± 7.5 C: 50.0 ± 9.6	Nil	Legume: Mixed (L, CP, B, P) 81 Control: No legumes	I: 49:33:17 C: 49:32:18	Isocaloric
								I: 22.9 ± 10.4 C: 18.2 ± 9.2	
Hermsdorff et al. 2011 [34]	P 8 wks	(15/15)	Obese, BMI: 32.5 ± 4.5	(17/13)	36.0 ± 8.0	Nil	Legumes: Mixed (L, CP, B, P) 113 Control: No legumes	I: 50:33:19 C: 51:31:19	Hypocaloric
								I: 26.0 ± 6.0 C: 18.0 ± 5.0	
Kazemi et al. 2018 [35]	P 16 wks	(30/31)	PCOS, BMI: I: 33.3 ± 9.0 C: 34.0 ± 9.8	(0/61)	I: 27.0 ± 4.6 C: 26.9 ± 4.4	Metformin (I/C, *n* = 18/20)	Legumes: Mixed (L, CP, B, P) ~244 Control: Legume free TLC	I: 57:30:16 C: 54:29:18	Isocaloric
								I: 33.3 ± 8.2 C: 24.5 ± 9.5	
Mollard et al. 2012 [36]	P 8 wks	(19/21)	Overweight/Obese, BMI: 32.8 ± 4.4	(11/29)	45.5 ± 6.3	Nil	Legumes: Mixed (L, CP, B, P) 128 Control: No legumes	I: 55:29:16 C: 51:32:17	Isocaloric
								I: 28.9 ± 9.1 C: 21.4 ± 6.4	
Nestel et al. 2004 [37]	C 6 wks	19	Healthy subjects, BMI 25.6 ± 3.2	(9/10)	56.6 ± 7.6	NR	Legumes: Chickpeas 140 Control: Wheat-based foods	I: 47:30:19 C: 44:31:19	Isocaloric
								I: 33.0 ± 8.0 C: 26.0 ± 13.0	
Saraf-Bank et al. 2016 [38]	C 6 wks	26	1° relatives w/T2DM, BMI: I: 28.7 ± 4.1 C: 29.0 ± 4.5	(12/14)	50.0 ± 6.6	Nil	Legumes: Mixed (L, B) 111 Control: No legumes	I: 66:20:16 C: 67:19:17	Isocaloric
								I: 38.4 ± 14.4 C: 32.3 ± 15.0	
Tonstad et al. 2014 [39] **	P 16 wks	(64/59)	Obese, T2DM (*n* = 35), BMI: I: 36.6 ± 3.8 C: 36.3 ± 4.1	(45/128) ^#^	I: 47.7 ± 10.2 C: 49.1 ± 11.2	Nil	Legumes: Beans (Mixed) 285 Control: Low CHO	I: 52:28:19 C: 32:42:27	Isocaloric
								I: 37.1 ± 21.9 C: 17.3 ± 10.2	
Winham et al. 2007 [40] (BB)	C 8 wks	23	Hyperlipidaemia, BMI: 27.4 ± 4.3	(10/13)	45.9 ± 10.5	NR	Legumes: Navy beans 95 Control: Carrots	I: 51:31:17 C: 51:33:17	Isocaloric
								I: 25.5 ± 17.1 C: 20.7 ± 16.2	

**Abbreviations:** Not Reported (NR); Weeks (wks); Grams per day (g/day); ^a^ Parallel (P), Cross-over (C); ^b^ Number of participants (*n*) Intervention (I), Control (C); ^c^ Waist Circumference (WC), Polycystic ovarian syndrome (PCOS), Metabolic Syndrome (MetSyn), Type 2 Diabetes Mellitus (T2DM), Body Mass Index (BMI) reported as kg/m^2^; ^d^ Male (M), Female (F); ^e^ Metformin, Oral agents for glycaemic control; ^f^ Lentils (L), Chickpeas (CP), Beans (B), Peas (P); ^g^ Reported as wet weight (1 g dry weight = 2.75 g wet weight, 1 mL = 0.76 g) [28]; ^h^ Macronutrient energy contribution (%E) (Carbohydrates: Fat: Protein); ^i^ Endpoint dietary fibre intake for legume intervention; ** Tonstad et al. 2014 included individuals with and without T2DM (T2DM ~20%), however, has been placed in comparison with those without diabetes based on mean baseline FBG and HbA1c measures; ^#^ Baseline data, endpoint NR. Winham et al. 2007 published two studies deemed eligible for inclusion within this review, one on individuals without diabetes using baked beans (BB) as intervention, and another on individuals with prediabetes.

**Table 3:** Characteristics of studies examining legume consumption in individuals with prediabetes.

Study	Design ^a^ and Duration	*n* (I/C) ^b^	Characteristics ^c^	(M/F) ^d^	Age (Years)	Anti-Diabetic Medication ^e^	Legume Type ^f^ Dose ^g^ (g/day) Control	%E (CHO: Fat: Pro) ^h^ Dietary Fibre ^i^ (g/day)	Energy Balance
Winhman et al. 2007 [41]	C 8 wks	16	Mild-mod IR, BMI: 27.8 ± 0.9	(7/9)	43.0 ± 12.0	NR	Legume: Pinto Beans 95 Control: Carrots	I: 51:32:15 C: 50:32:17	Isocaloric
								I: 23.0 ± 15.6 C: 21.0 ± 15.6	
Winham et al. 2007 [41]	C 8 wks	16	Mild-mod IR, BMI: 27.8 ± 0.9	(7/9)	43.0 ± 12.0	NR	Legume: Black-eyed peas 95 Control: Carrots	I: 53:31:16 C: 50:32:17	Isocaloric
								I: 19.0 ± 15.7 C: 21.0 ± 15.6	

**Abbreviations:** Not Reported (NR); Weeks (wks); Grams per day (g/day); ^a^ Parallel (P), Cross-over (C); ^b^ Number of participants (*n*) Intervention (I), Control (C); ^c^ Insulin Resistant (IR), defined as (FBI >15 µU/mL), Body Mass Index (BMI) reported as kg/m^2^; ^d^ Male (M), Female (F); ^e^ Metformin, Oral agents for glycaemic control; ^f^ Lentils (L), Chickpeas (CP), Beans (B), Peas (P), ^g^ Reported as wet weight (1 g dry weight = 2.75 g wet weight, 1 mL = 0.76 g) [28]; ^h^ Macronutrient energy contribution (%E) (Carbohydrates: Fat: Protein); ^i^ Endpoint dietary fibre intake for legume intervention.

**Table 4:** The Grading of Recommendations, Assessment, Development and Evaluations (GRADE) summary of findings: fasting blood glucose (FBG) and glycosylated haemoglobin (HbA1c) in individuals with type 2 diabetes mellitus (T2DM).

Outcome, *n* Studies, (I/C)	Criteria for Downgrading Quality	Assessment and Justification	Quality of Evidence ^a^
FBG, 4 studies, (141/145)	*Initial Quality*	High; Randomised Controlled Trials only	Very Low 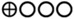
	Risk of bias	Evidence not downgraded; Two studies were rated as ‘low RoB’, and two were rated as ‘some concerns’. Limitations were not serious	
	Inconsistency	Evidence not downgraded; Visual inspection identified consistency within size of effect	
	Indirectness	Downgrade by one level; Population (T2DM; direct), intervention (Legume dose (g/day) varied between studies), comparisons (control interventions varied between studies), outcomes (FBG; direct)	
	Imprecision	Downgrade by one level; Insufficient sample size according to OIS	
	Publication bias	Downgrade by one level; Grey literature sources were not included in defined search strategy	
HbA1c, 3 studies, (98/102)	*Initial Quality*	High; Randomised Controlled Trials only	Very Low 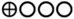
	Risk of bias	Evidence not downgraded; One study rated as ‘low RoB’, one ‘some concerns’ and one ‘high’ due to absence of wash-out period, contribution of study was small. Limitations were not serious	
	Inconsistency	Evidence not downgraded; Visual inspection identified consistency within size of effect	
	Indirectness	Downgrade by one level; Population (T2DM; consistent), intervention (Legume dose (g/day) varied between studies), comparisons (control interventions varied between studies), outcomes (HbA1c; direct)	
	Imprecision	Downgrade by one level; Insufficient sample size according to OIS	
	Publication bias	Downgrade by one level; Grey literature sources were not included in defined search strategy	

**Abbreviations:** Intervention (I); Control (C); Risk of Bias (RoB) as determined by the Revised Cochrane Risk of Bias Tool; ^a^ Quality of evidence grades: High, Moderate, Low, Very Low, Optimal Information Size (OIS) according to =0.05, =0.2 [43].

**Table 5:** Effects of legume consumption on markers of glycaemic control in individuals with diabetes mellitus.

**Study**	***n* (I/C)**	**FBG Baseline (mmol/L)**	**FBG Endpoint (mmol/L)**	**Statistical Significance**	
				**Within-Group**	**Between-Group**
Hassanzadeh-Rostami et al. 2019 [23]	I (*n* = 20) C (*n* = 23)	7.99 (6.37, 8.82) * 9.60 (6.38, 13.1)	7.38 (6.22, 8.44) 8.21 (6.60, 9.64)	NS NS	NS
Hosseinpour-Niazi et al. 2015 [24]	I (*n* = 31) C (*n* = 31)	7.94 ± 3.09 8.19 ± 2.97	6.35 ± 2.26 7.11 ± 2.75	*p* < 0.05 *p* < 0.05	*p* < 0.001
Jenkins et al. 2012 [25]	I (*n* = 60) C (*n* = 61)	7.83 ± 1.30 7.44 ± 1.52	7.33 ± 1.30 7.05 ± 1.29	*p* < 0.05 NS	*p* = 0.01
Shams et al. 2010 [26]	I (*n* = 30) C (*n* = 30)	8.56 ± 0.82 8.58 ± 0.69	8.43 ± 0.70 8.50 ± 0.57	*p* < 0.05 NS	*p* < 0.05
**Study**	***n* (I/C)**	**FBI Baseline (pmol/L)**	**FBI Endpoint (pmol/L)**	**Statistical Significance**	
				**Within-Group**	**Between-Group**
Hassanzadeh-Rostami et al. 2019 [23]	I (*n* = 20) C (*n* = 23)	15.7 (9.10, 35.4) * 8.20 (5.60, 11.3)	13.9 (8.20, 23.8) 7.50 (4.90, 10.6)	NS NS	*p* = 0.02
Hosseinpour-Niazi et al. 2015 [24]	I (*n* = 31) C (*n* = 31)	48.6 ± 20.0 45.0 ± 23.4	27.0 ± 10.0 36.0 ± 16.7	*p* < 0.05 *p* < 0.05	*p* = 0.006
**Study**	***n* (I/C)**	**HbA1c Baseline (%)**	**HbA1c Endpoint (%)**	**Statistical Significance**	
				**Within-Group**	**Between-Group**
Hassanzadeh-Rostami et al. 2019 [23]	I (*n* = 20) C (*n* = 23)	7.70 (7.00, 9.10) * 9.50 (8.10, 11.3)	7.60 (7.00, 9.30) 9.00 (7.70, 11.5)	NS NS	*p* = 0.04
Jenkins et al. 2012 [25]	I (*n* = 60) C (*n* = 61)	7.40 ± 0.58 7.20 ± 0.59	6.90 ± 0.58 6.90 ± 0.39	*p* < 0.05 NS	*p* < 0.01
Simpson et al. 1981 [27] (T2DM)	I (*n* = 18) C (*n* = 18)	NR NR	8.60 ± 1.60 9.60 ± 2.30	NR NR	*p* < 0.02
Simpson et al. 1981 [27] (T1DM)	I (*n* = 9) C (*n* = 9)	NR NR	9.80 ± 1.80 10.0 ± 2.30	NR NR	NS
**Study**	***n* (I/C)**	**2-h PPG Baseline (mmol/L)**	**2-h PPG Endpoint (mmol/L)**	**Statistical Significance**	
				**Within-Group**	**Between-Group**
Simpson et al. 1981 [27] (T2DM)	I (*n* = 18) C (*n* = 18)	NR NR	8.10 ± 1.60 9.10 ± 2.30	NR NR	*p* < 0.05
Simpson et al. 1981 [27] (T1DM)	I (*n* = 9) C (*n* = 9)	NR NR	9.10 ± 3.30 12.2 ± 3.40	NR NR	*p* < 0.02

**Abbreviations**: Not reported (NR); Not significant (NS) according to Study *p*-value; Fasting Blood Glucose (FBG); Fasting Blood Insulin (FBI); Glycosylated haemoglobin, % value of total haemoglobin (HbA1c); 2-h Postprandial Glucose (2-h PPG); Type 2 Diabetes Mellitus (T2DM); Type 1 Diabetes Mellitus (T1DM); Number of participants (*n*); Intervention (I); Control (C); * Reported baseline and outcome values displayed as mean (25th percentile, 75th percentile).

**Table 6:** Effects of legume consumption on fasting blood glucose (FBG) in individuals without diabetes mellitus.

Study	*n* (I/C)	FBG Baseline (mmol/L)	FBG Endpoint (mmol/L)	Statistical Significance	
				Within-Group	Between-Group
Abete et al. 2009 [29]	I (*n* = 8) C (*n* = 10)	NR NR	NR NR	*p* < 0.05 NS	NS
Abeysekara et al. 2012 [30]	I (*n* = 87) C (*n* = 87)	4.37 ± 1.40 4.47 ± 1.92	4.39 ± 1.36 4.17 ± 1.51	NS NS	NS
Alizadeh et al. 2014 [31]	I (*n* = 17) C (*n* = 17)	5.09 ± 1.33 5.12 ± 1.46	5.12 ± 1.43 5.21 ± 1.43	NS NS	NS
Crujeiras et al. 2007 [32]	I (*n* = 15) C (*n* = 15)	NR NR	NR NR	NS NS	NS
Gravel et al. 2010 [33]	I (*n* = 60) C (*n* = 54)	5.30 ± 0.64 5.20 ± 0.54	5.28 ± 0.69 5.34 ± 0.61	NS NS	NS
Hermsdorff et al. 2011 [34]	I (*n* = 15) C (*n* = 15)	5.17 ± 0.32 5.13 ± 0.53	5.13 ± 0.29 4.98 ± 0.37	NS NS	NS
Kazemi et al. 2018 [35]	I (*n* = 30) C (*n* = 31)	5.00 ± 1.50 5.60 ± 1.40	4.60 ± 1.30 4.80 ± 1.60	*p* < 0.01 *p* < 0.01	NS
6-month follow-up	I (*n* = 16) C (*n* = 16)	5.30 ± 1.70 5.50 ± 1.50	4.90 ± 0.20 5.30 ± 0.90	NS NS	NS
12-month follow-up	I (*n* = 12) C (*n* = 13)	5.20 ± 1.10 5.50 ± 1.40	4.90 ± 0.60 5.30 ± 0.50	NS NS	NS
Mollard et al. 2012 [36]	I (*n* = 19) C (*n* = 21)	NR NR	NR NR	NS NS	NS
Nestel et al. 2004 [37]	I (*n* = 19) C (*n* = 19)	5.20 ± 0.40 5.20 ± 0.40	4.90 ± 0.40 5.10 ± 0.50	NS NS	NS
Saraf-Bank et al. 2016 [38]	I (*n* = 26) C (*n* = 26)	5.35 ± 2.08 5.28 ± 2.34	5.38 ± 2.28 5.42 ± 2.28	NS NS	NS
Tonstad et al. 2014 [39]	I (*n* = 64) C (*n* = 59)	5.60 ± 1.90 5.90 ± 1.90	5.30 ± 1.60 5.60 ± 1.50	NS NS	NS
12-month follow-up	I (*n* = 30) C (*n* = 24)	5.30 ± 1.10 5.30 ± 0.80	5.30 ± 0.90 5.30 ± 0.80	NS NS	NS
Winham et al. 2007 [40] (BB)	I (*n* = 23) C (*n* = 23)	5.61 ± 1.81 5.77 ± 2.21	5.49 ± 1.81 5.77 ± 1.81	NS NS	NS

**Abbreviations:** Not reported (NR); Not significant (NS) according to study *p*-value; Fasting Blood Glucose (FBG); Number of participants (*n*); Intervention (I); Control (C). Winham et al. 2007 published two studies deemed eligible for inclusion within this review, one on individuals without diabetes using baked beans (BB) as intervention, and another on individuals with prediabetes.

**Table 7:** Effects of legume consumption on fasting blood insulin (FBI) in individuals without diabetes mellitus.

Study	*n* (I/C)	FBI Baseline (pmol/L)	FBI Endpoint (pmol/L)	Statistical Significance	
				Within-Group	Between-Group
Abete et al. 2009 [29]	I (*n* = 8) C (*n* = 10)	NR NR	NR NR	NS NS	NS
Abeysekara et al. 2012 [30]	I (*n* = 87) C (*n* = 87)	75.7 ± 74.0 82.6 ± 72.2	74.8 ± 71.0 71.3 ± 107	NS NS	NS
Alizadeh et al. 2014 [31]	I (*n* = 17) C (*n* = 17)	113 ± 27.2 109 ± 69.3	114 ± 37.1 107 ± 47.0	NS NS	NS
Crujeiras et al. 2007 [32]	I (*n* = 15) C (*n* = 15)	NR NR	NR NR	NS NS	NS
Gravel et al. 2010 [33]	I (*n* = 60) C (*n* = 54)	89.3 ± 44.8 81.2 ± 37.3	88.7 ± 43.4 88.3 ± 51.4	NS NS	NS
Hermsdorff et al. 2011 [34]	I (*n* = 15) C (*n* = 15)	45.0 ± 22.8 63.0 ± 60.0	35.4 ± 24.0 49.2 ± 25.8	NS NS	NS
Kazemi et al. 2018 [35]	I (*n* = 30) C (*n* = 31)	84.0 ± 68.4 94.2 ± 74.4	60.0 ± 46.2 76.2 ± 61.8	*p* < 0.01 *p* < 0.01	NS
6-month follow up	I (*n* = 16) C (*n* = 16)	81.0 ± 76.8 87.6 ± 21.6	79.8 ± 67.2 100 ± 54.6	NS NS	NS
12-month follow-up	I (*n* = 12) C (*n* = 13)	97.8 ± 91.8 108 ± 104	84.6 ± 57.6 99.0 ± 60.0	*p* < 0.02 *p* < 0.02	NS
Mollard et al. 2012 [36]	I (*n* = 19) C (*n* = 21)	NR NR	NR NR	NS NS	NS
Nestel et al. 2004 [37]	I (*n* = 19) C (*n* = 19)	39.6 ± 21.6 39.6 ± 21.6	47.4 ± 27.0 49.2 ± 28.2	NS NS	NS
Winham et al. 2007 [40] (BB)	I (*n* = 23) C (*n* = 23)	126 ± 57.6 156 ± 143.9	120 ± 57.6 126 ± 57.6	NS NS	NS

**Abbreviations:** Not reported (NR); Not significant (NS) according to study *p*-value; Fasting Blood Insulin (FBI); Number of participants (*n*); Intervention (I); Control (C). Winham et al. 2007 published two studies deemed eligible for inclusion within this review, one on individuals without diabetes using baked beans (BB) as intervention, and another on individuals with prediabetes.

## Supplementary Materials

The following are available at https://www.mdpi.com/2072-6643/12/7/2123/s1; Table S1: PICO Framework, Table S2: Search Terms (Medline), Figure S1: Results of Risk of Bias Assessment, Table S3: GRADE summary of findings for FBG, FBI, HOMA-IR and HbA1c for individuals without diabetes mellitus.
