# Supplementary material for: The Effects of Legume Consumption on Markers of Glycaemic Control in Individuals with and without Diabetes Mellitus: A Systematic Literature Review of Randomised Controlled Trials
Source: Nutrients. 2020 Jul 17;12(7):2123. doi: 10.3390/nu12072123 (PMC7400945; doi:10.3390/nu12072123)
Supplement: Supplementary file 1 [file nutrients-12-02123-s001.pdf]

Supplementary Materials

Table 1. PICO framework: Is there an effect of legume consumption on markers of glycaemic control in those with diabetes mellitus (T2DM, T1DM, GDM), without diabetes mellitus, or those with prediabetes?

PICO Categories	Included	Excluded
**Population**	Adults (aged ≥18 years); Without diabetes, With prediabetes (defined by author), Type 2 Diabetes Mellitus (T2DM), Type 1 Diabetes Mellitus (T1DM), or Gestational Diabetes Mellitus (GDM)	Children (aged <18 years); Studies on those with Diabetes Insipidus
**Intervention**	Legume-only intervention, to investigate the independent effect; ≥6 weeks duration; Including: chickpeas, beans (kidney, pinto, black, cannellini, white, fava, adzuki, borlotti, flageolet, lima, mung), peas (black-eyed, blue, maple, white, dun), lentils (green, red, yellow, French), lupin, or non-oil seed pulses.	Trial arms not randomised; Studies including peanuts, soybeans or soy products; Combined interventions including legumes; Dietary patterns that encompass legumes; Legume provision in extract form
**Control/ Comparator**	Legume intervention diet compared with a control diet without legumes	Comparator including soybeans
**Outcome**	Changes between baseline and follow-up in markers of glycaemic control including changes in glycated haemoglobin (HbA1c), fasting blood glucose (FBG), fasting blood insulin (FBI), homeostatic model assessment of insulin resistance (HOMA-IR) or 2-hour postprandial glucose (2-h PPG)	Qualitative measures

Table 2. Search terms (MEDLINE).

1.	legume*.tw.
2.	fabaceae.tw.
3.	lentil*.tw.
4.	chickpea*.tw.
5.	peas.tw.
6.	pea.tw.
7.	pulses.tw.
8.	bean*.tw.
9.	lupin.tw.
10.	fava.tw.
11.	faba.tw.
12.	pinto.tw.
13.	adzuki.tw.
14.	borlotti.tw.
15.	cannellini.tw.
16.	flageolet.tw.
17.	lima bean*.tw.
18.	mung.tw.
19.	black-eyed.tw.
20.	exp fabaceae/
21.	1 or 2 or 3 or 4 or 5 or 6 or 7 or 8 or 9 or 10 or 11 or 12 or 13 or 14 or 15 or 16 or 17 or 18 or 19 or 20
22.	Randomized Controlled Trials as Topic/
23.	Randomized Controlled Trial/
24.	Random Allocation/
25.	Double Blind Method/
26.	Single blind method/
27.	clinical trial/
28.	clinical trial, phase i.pt.
29.	clinical trial, phase ii.pt.
30.	clinical trial, phase iii.pt.
31.	clinical trial, phase iv.pt.
32.	controlled clinical trial.pt.
33.	randomized controlled trial.pt.
34.	multicenter study.pt.
35.	clinical trial.pt.
36.	exp Clinical Trials as topic/
37.	or/22-36
38.	(clinical adj trial$).tw.
39.	((singl$ or doubl$ or treb$ or tripl$) adj (blind$3 or mask$3)).tw.
40.	PLACEBOS/
41.	placebo$.tw.
42.	randomly allocated.tw.
43.	(allocated adj2 random$).tw.
44.	or/38-43
45.	37 or 44

46. case report.tw.
47. letter/
48. historical article/
49. or/46-48
50. 45 not 49
51. Glucose metabolism disorders/
52. Diabetes Mellitus/
53. exp Diabetes Mellitus, Type 2/
54. exp Diabetes Mellitus, Type 1/
55. Diabetes, Gestational/
56. Healthy Volunteers/
57. exp Insulin Resistance/
58. exp Glycated Haemoglobin A/
59. insulin, regular, human/
60. Insulin/
61. exp Postprandial Period/
62. exp glycaemic load/
63. exp glycaemic index/
64. exp Fructosamine/
65. exp Hyperglycaemia/
66. exp Prediabetic State/
67. exp Blood Glucose/
68. exp Glucose Tolerance Test/
69. exp Glucose Intolerance/
70. glucose.tw.
71. insulin.tw.
72. postprandial.tw.
73. glyc?emi*.tw.
74. hyperglyc?emi*.tw.
75. HbA1c.tw.
76. (fasting adj2 (insulin or glucose)).tw.
77. insulin resistance.tw.
78. oral glucose tolerance.tw.
79. fructosamine.tw.
80. OGTT.tw.
81. HOMA-IR.tw.
82. Glycated albumin.tw.
83. prediabet*.tw.
84. diabet*.tw.
85. normoglyc?emi*.tw.
86. T2DM.tw.
87. T2D.tw.
88. T1DM.tw.
89. T1D.tw.
90. GDM.tw.
91. IFG.tw.
92. IGT.tw.

93. (impaired adj2 glucose).tw.
94. ((glycated or glycosylated) adj1 h?emoglobin*).tw.
95. gestational diabetes.tw.
96. blood glucose.tw.
97. Homeostatic model assessment.tw.
98. NIDDM.tw.
99. IDDM.tw.
100. 51 or 52 or 53 or 54 or 55 or 56 or 57 or 58 or 59 or 60 or 61 or 62 or 63 or 64 or 65 or 66 or 67 or 68 or 69 or 70 or 71 or 72 or 73 or 74 or 75 or 76 or 77 or 78 or 79 or 80 or 81 or 82 or 83 or 84 or 85 or 86 or 87 or 88 or 89 or 90 or 91 or 92 or 93 or 94 or 95 or 96 or 97 or 98 or 99
101. 21 and 50 and 100
102. Animal/
103. Human/
104. 102 and 103
105. 102 not 104
106. 101 not 105

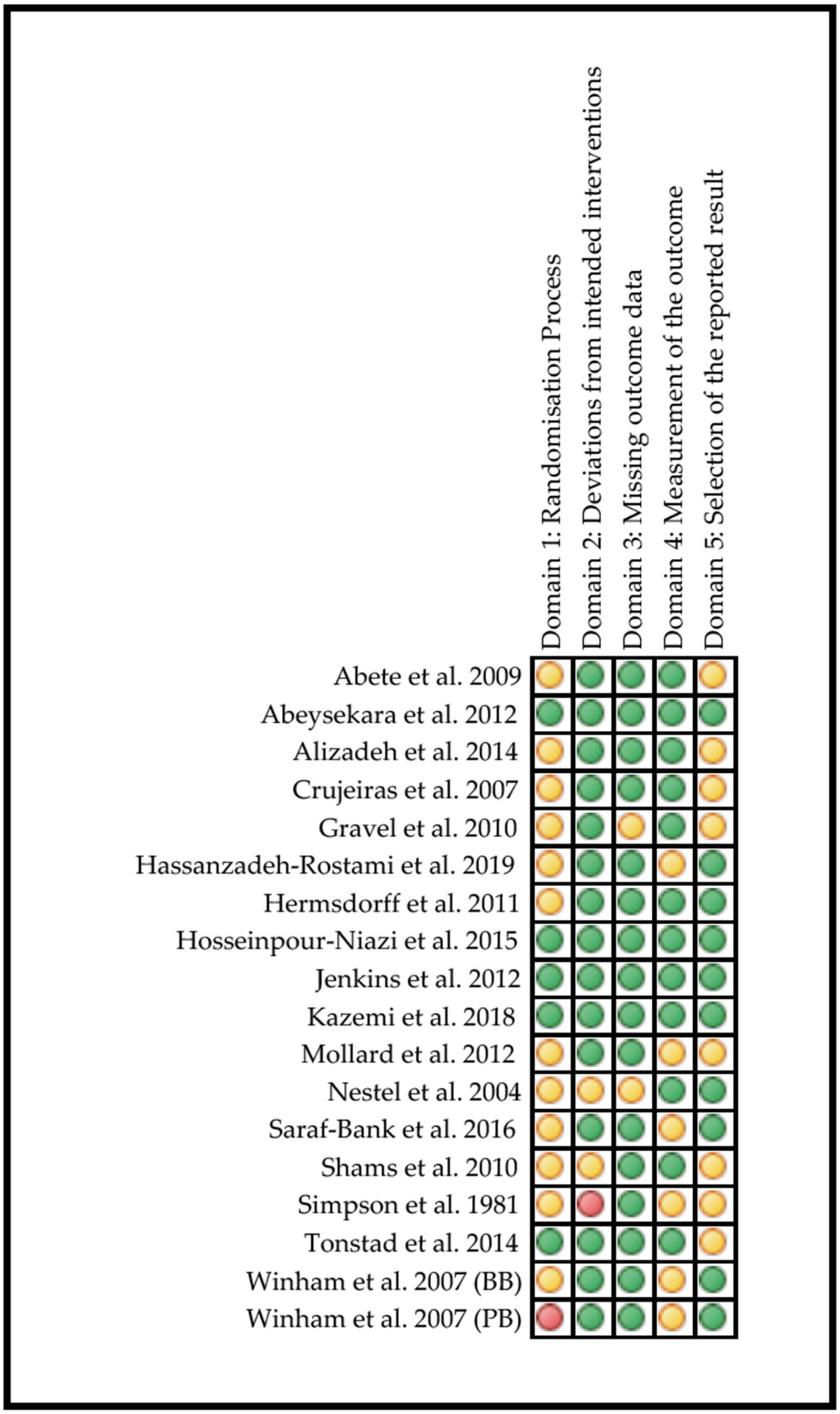

Figure 1. Results of the risk of bias assessment.

Table 3. GRADE summary of findings: FBG, FBI, HOMA-IR, and HbA1c in individuals without diabetes mellitus.

Outcome, n studies, (I/C)	Criteria for downgrading quality	Assessment and justification	Quality of evidence[a]
FBG, 12 studies, (383/430)	***Initial Quality***	High; Randomised Controlled Trials only	Very Low ⊕○○○
	**Risk of bias**	Not downgraded; Two studies were rated as 'low RoB, and 10 were rated as 'some concerns', limitations were not serious	
	**Inconsistency**	Not downgraded; Visual inspection identified consistency within size of effect	
	**Indirectness**	Downgrade one level; Population (comorbidities present), intervention (Legume dose (g/d) varied), comparisons (controls varied), outcomes (FBG; direct)	
	**Imprecision**	Downgrade one level; Seven sample sizes insufficient according to OIS, three did not report raw data therefore could not be determined	
	**Publication bias**	Downgrade one level; Grey literature sources were not included	
FBI, 10 studies, (293/290)	***Initial Quality***	High; Randomised Controlled Trials only	Very Low ⊕○○○
	**Risk of bias**	Not downgraded; Two studies rated as 'low RoB', eight as 'some concerns', limitations were not serious	
	**Inconsistency**	Not downgraded; Visual inspection identified consistency within size of effect	
	**Indirectness**	Downgrade one level; Population (comorbidities present), intervention (Legume dose (g/d) varied), comparisons (controls varied), outcomes (FBI; direct)	
	**Imprecision**	Downgrade one level; One sample size sufficient according to OIS, three did not report raw data, remaining seven sample sizes insufficient	
	**Publication bias**	Downgrade one level; Grey literature sources were not included	
HOMA-IR 7 studies, (131/136)	***Initial Quality***	High; Randomised Controlled Trials only	Very Low ⊕○○○
	**Risk of bias**	Not downgraded; One study rated as 'low RoB', six as 'some concerns', limitations were not serious	
	**Inconsistency**	Not downgraded; Visual inspection identified consistency within size of effect	

	**Indirectness**	Downgrade one level; Population (comorbidities present), intervention (Legume dose (g/d) varied), comparisons (controls varied), outcomes (HOMA-IR; direct)	
	**Imprecision**	Downgrade one level; One sample size sufficient according to OIS, two did not report raw data, remaining four sample sizes insufficient	
	**Publication bias**	Downgrade one level; Grey literature sources were not included	
HbA1c, 5 studies, (164/160)	***Initial Quality***	High; Randomised Controlled Trials only	Very Low ⊕○○○
	**Risk of bias**	Not downgraded; One study rated as 'low RoB', four as 'some concerns', limitations were not serious	
	**Inconsistency**	Not downgraded; Visual inspection identified consistency within size of effect	
	**Indirectness**	Downgrade one level; Population (comorbidities present), intervention (Legume dose (g/d) varied), comparisons (controls varied), outcomes (HbA1c; direct)	
	**Imprecision**	Downgrade one level; sample sizes insufficient according to OIS	
	**Publication bias**	Downgrade one level; Grey literature sources were not included	

Abbreviations: Intervention (I), Control (C), Risk of Bias (RoB) as determined by the Revised Cochrane Risk of Bias Tool, [a]Quality of evidence grades: High, Moderate, Low, Very Low, Optimal Information Size (OIS) according to $\alpha = 0.05$, $\beta = 0.2$.
